# Surgical management of anterior clinoidal meningiomas: consensus statement on behalf of the EANS skull base section

**DOI:** 10.1007/s00701-021-04964-3

**Published:** 2021-08-16

**Authors:** D. Starnoni, C. Tuleasca, L. Giammattei, G. Cossu, M. Bruneau, M. Berhouma, J. F. Cornelius, L. Cavallo, S. Froelich, E. Jouanneau, T. R. Meling, D. Paraskevopoulos, H. Schroeder, M. Tatagiba, I. Zazpe, A. Sufianov, M. E. Sughrue, Ari G. Chacko, V. Benes, P. González-Lopez, Pierre-Hugues Roche, Marc Levivier, Mahmoud Messerer, Roy T. Daniel

**Affiliations:** 1 Department of Neurosurgery, University Hospital of Lausanne and Faculty of Biology and Medicine, University of Lausanne, Rue du Bugnon 46, CH-1011 Lausanne, Switzerland; 2 Signal Processing Laboratory (LTS 5), Ecole Polytechnique Fédérale de Lausanne (EPFL), Lausanne, Switzerland; 3 Department of Neurosurgery, Lariboisière Hospital, Paris, France; 4 Department of Neurosurgery, Vrije Universiteit Brussel (VUB), Universitair Ziekenhuis Brussel (UZ Brussel), Laarbeeklaan 101, 1090 Brussels, Belgium; 5 Department of Neurosurgery, Hôpital Neurologique Pierre Wertheimer, Lyon, France; 6 Department of Neurosurgery, Medical Faculty, Heinrich-Heine-University Düsseldorf, Düsseldorf, Germany; 7 Department of Neurosurgery, University Hospital of Naples Federico II, Napoli, Italy; 8 Department of Neurosurgery, University Hospital of Geneva, Geneva, Switzerland; 9 Department of Neurosurgery, Barts Health NHS Trust, St. Bartholomew’s and The Royal London Hospital, London, UK; 10 Department of Neurosurgery, University Medicine Greifswald, Greifswald, Germany; 11 Department of Neurosurgery, Eberhard Karls University of Tübingen, Tübingen, Germany; 12 Department of Neurosurgery, Complejo Hospitalario de Navarra, Pamplona, Spain; 13 Federal Centre of Neurosurgery, Tyumen, Russian Federation; 14 I.M. Sechenov First Moscow State Medical University, Moscow, Russian Federation; 15 Centre for Minimally Invasive Neurosurgery, Prince of Wales Private Hospital, Sydney, Australia; 16 Department of Neurological Sciences, Christian Medical College, Vellore, Tamil Nadu India; 17 Department of Neurosurgery, First Medical Faculty, Military University Hospital and Charles University, Prague, Czech Republic; 18 Department of Neurosurgery, Hospital General Universitario de Alicante, Institute for Health and Biomedical Research of Alicante (ISABIAL), Alicante, Spain; 19 Department of Neurosurgery, CHU North Hospital, Aix-Marseille University, Marseille, France

**Keywords:** Anterior clinoid process, Clinoidal meningiomas, Combined management, Medial third sphenoid wing meningiomas, Microsurgery, Radiosurgery

## Abstract

**Background:**

The optimal management of clinoidal meningiomas (CMs) continues to be debated.

**Methods:**

We constituted a task force comprising the members of the EANS skull base committee along with international experts to derive recommendations for the management of these tumors. The data from the literature along with contemporary practice patterns were discussed within the task force to generate consensual recommendations.

**Results and conclusion:**

This article represents the consensus opinion of the task force regarding pre-operative evaluations, patient’s counselling, surgical classification, and optimal surgical strategy. Although this analysis yielded only Class B evidence and expert opinions, it should guide practitioners in the management of patients with clinoidal meningiomas and might form the basis for future clinical trials.

## Introduction

Clinoidal meningiomas remain a challenging pathology because of their intimate relationship to vital neurovascular structures [43, 68]. Among skull base lesions, these are tumors that are still associated with high surgical morbidity and recurrence, next only to petroclival meningiomas [5]. Clinoidal meningiomas were clearly defined for the first time in 1938 by Cushing, who identified a distinct subgroup of medial third meningiomas from the larger cohort of sphenoidal ridge meningiomas [11]. These lesions account for less than half of sphenoidal ridge meningiomas and, based on their specific features (origin, dural attachment, growth pattern, neurovascular relationships) should be considered specific entities, distinct from spheno-cavernous meningiomas, though this distinction can occasionally be difficult [2, 74]. Pure clinoidal meningiomas have their epicenter on the anterior clinoid process (ACP) and tend to grow upward with a small pedicle toward the suprasellar area and the Sylvian fissure [55]. Their peculiar site of origin, inferolateral to the ACP and intimately related with the vessels supplying the optic apparatus, accounts for the poorer visual outcome and higher rate of subtotal resection (STR) compared with the more medially situated meningiomas (planum, diaphragma, and tuberculum meningiomas) [38]. Clinoidal meningiomas usually present with headache and visual disturbances but can become symptomatic with additional cranial neuropathies such as diplopia and facial hypoesthesia through cavernous sinus or superior orbital fissure invasion [19]. The main indication for surgery in these tumors is cranial neuropathy due to tumoral compression and/or radiological growth (in asymptomatic patients). Surgical management of clinoidal meningiomas differs largely between surgical series, primarily because they are often grouped with other sphenoid ridge meningiomas. The main aim of this consensus paper was to define the current optimal surgical management of pure clinoidal meningiomas.

## Methods

The EANS skull base section was created in October 2017, and its board decided to review the state of the art with respect to some controversial topics in the field, in order to generate recommendations from a European perspective. The optimal management of clinoidal meningiomas, especially the surgical strategy, continues to be debated. This work represents the consensually derived opinion and recommendations of the EANS skull base section board with the valuable participation of invited renowned experts in this field based on an earlier published systematic review and meta-analysis of studies in literature (Table 1), followed by formal discussions within the group [19]. The methods to identify, select, and critically appraise relevant research from previously published studies were detailed in our previous publication of the meta-analysis on clinoidal meningiomas [19]. Similar to the guidelines published by the EANS skull base section on other cranial base pathologies, the quality of evidence was assessed using the Grading of Recommendations, Assessment, Development and Evaluation (GRADE) Working Group system [3]. If randomized blinded trials or prospective matched-pair cohort studies were identified, the recommendations were level A or B. For controlled non-randomized trials or uncontrolled studies, the recommendations were level C or “expert opinion,” respectively. The results of the meta-analysis and the systematic review of literature were discussed within the task force to generate recommendations in a consensual manner.

**Table 1:** Review of microsurgical series reporting on anterior clinoidal meningiomas

Reference	N	Adopted classification	Presenting visual impairment (%)	Postoperative visual status (%)	Major vessels encasement (%)	Optic canal invasion (%)	Cavernous sinus invasion (%)	Gross total resection (%)	Clinoidectomy (%)	Resection of the intracavernous portion (%)
Al-Mefty 1990 [2]	24	3 (12.5%) Al- Mefty group I 19 (79.2%) Al- Mefty group II 2 (8.3%) Al- Mefty III	20 (84)	2 (10) improved 17 (85) stable 1 (5) deteriorated	14 (58.3)	NR	9 (37.5)	18 (54.5)	24 (100)/extradural	NR
Risi et al. 1994 [62]	34	Angiography-based classification	20 (58.8)	6 (31.6) improved 7 (36.8) stable 6 (31.6) deteriorated*	NR	NR	15 (44.1)	18 (54.5)	34 (100)/extradural	8 (61.5)
Puzzilli et al. 1999 [60]	33	6 (18.2%) Al- Mefty group I 22 (66.7%) Al- Mefty group II 5 (15.1%) Al- Mefty group III	15 (45.5)	NR	18 (54.5)	12 (36.4)	13 (39.4)	18 (54.5)	NR	8 (61.5)
Lee et al. 2001 [34]	15	NA	8 (53.3)	6 (75) improved 2 (25) stable	NR	5 (33.3)	2 (13,3)	13 (86.7)	13 (86.7)/extradural	1 (50)
Tobias et al. 2003 [73]	26	3 (11.5%) Al- Mefty group I 19 (73%) Al- Mefty group II 2 (7.6%) Al- Mefty group III	14 (53.8)	10 (71.4) improved 4 (28.6) stable	NR	NR	6 (23)	20 (77)	24 (92.3)/extradural	NR
Nakamura et al. 2006 [49]	108	39 (36.2%) group I (no CS invasion) 69 (63.8%) group II (CS invasion)	68 (63)	21/45 (46.6) improved 18/45 (40) stable 6/45 (13) deteriorated*	76 (70.3)	NR	69 (63.8)	46 (42.6)	NR/intradural	10 (14.4)
Cui et al. 2007 [10]	26	4 (15.3%) Al- Mefty group I 22 (84.6%) Al- Mefty group II	22 (84.6)	16 (72.7) improved 6 (27.3) stable	NR	NR	4 (17.4)	16 (61.5)	26 (100)/extradural	0
Pamir et al. 2008 [55]	43	2 (4.7%) Al- Mefty group I 38 (88.4%) Al- Mefty group II 3 (6.9%) Al- Mefty group III	26 (60.5)	22 (84.6) improved 4 (15.3) stable	13 (30.2	NR	1 (2.3)	39 (90.7)	40 (93)/intradural	0
Sade and Lee 2008 [65]	52	NA	24 (46)	17 (77) improved 5 (23) stable*	NR	19 (36)	NR	37 (71.2)	47 (90.4)/extradural	0
Bassiouni et al. 2009 [5]	106	NA	52 (49.1)	21 (40.4) improved 24 (46.2) stable 7 (13.5) deteriorated	51 (48.1)	16 (15)	31 (29)	62 (58.5)	23 (22)/intradural	0
Romani et al. 2011 [63]	73	20 (27.4%) small < 2 cm 32 (43.8%) Intermediate 2 e4 cm 21 (28.7%) large > 4 cm	39 (53)	11 (28.2) improved 24 (61.5) stable 4 (10.3) deteriorated	53 (57)	10 (14)	17 (23.2)	57 (78)	21 (28.7)/intradural	NR
Liu et al. 2012 [37]	127	NA	74 (58.2)	42 (56.8) improved 30 (40.5) stable 2 (2.7) deteriorated	56 (44.1)	10 (7.9)	60 (47.2)	7 (31.8)	NR/extradural	NR
Attia et al. 2012 [4]	22	NA	19 (86.4)	12(66.7) improved 4 (22.2) stable 2 (11.1) deteriorated*	20 (90)	17 (77.3)	13 (59.1)	7 (31.8)	19 (86.3)/extradural	0
Nagata et al 2013 [48]	23	Angiography-based venous drainage classification	15 (65)	10 (66) improved 5 (33) stable	NR	NR	8 (34.7)	9 (39)	NR/extradural	0
Mariniello et al. 2013 [42]	46	11 (24%) Al- Mefty group I 28 (60.8%) Al- Mefty group II 7 (15.2%) Al- Mefty group III	30 (65.2)	17 (56.7) improvement 12 (40) stable 1 (3.3) deteriorated	NR	10 (22)	7 (15)	39 (84.8)	20 (43.5)/extradural	0
Czernicki et al. 2015 [12]	30	6 (20%) Al- Mefty group I 20 (66.7%) Al- Mefty group II 4 (13.3%) Al- Mefty group III	18 (60)	6 (33.3) improved 11 (61.1) stable 1 (5.6) deteriorated .2 new worsening	5 (16.6)	NR	6 (20)	19 (63.3)	30 (100)/extradural	0
Sughrue et al. 2015 [68]	29	NA	20 (69)	5 (25) improved 11 (55) stable 4 (20) deteriorated	14 (48.3)	15 (51.7)	7 (24.1)	6 (20.7)	NR/extradural	0
Nanda et al. 2016 [50]	36	36 (100) grade I	32 (89)	9 (28) improved 20 (63) stable 3 (9) deteriorated	16 (44)	13 (36)	14 (38.9)	27 (75)	NR/intradural	0
Verma et al. 2016 [75]	78	76 (97.4) grade I 1 (1.3) grade II 1 (1.3) grade III	58 (74.3)	12 (20.7) improved 39 (67.2) stable 7 (12.1) deteriorated	43 (55.1)	NR	21 (26.9)	52 (66.7)	78 (100)/extradural	NR
Kim et al. 2017 [30]	59	50 (84.7) grade I 7 (11.9) grade II 2 (3.4) grade III	17 (28.8)	NR	45 (76.3)	2 (3.4)	8 (13.6)	36 (60)	59 (100)/extradural	0
Talacchi et al. 2018 [70]	60	3 groups according to number of encased vessels	31 (51.7)	16 (51.6) improvement 15 (48.4) stable	30 (50)	NR	18 (30)	36 (60)	NR/intradural	0
Giammattei et al. 2019 [19]	18	6 (33.3%) Al- Mefty I 10 (55.6%) Al- Mefty group II 2 (11.1) Al- Mefty group III	12 (66.6)	5 (41.6) improved 6 (50) stable 1 (8.3) deteriorated	9 (50)	8 (44.4)	6 (33.3)	12 (67)	18 (100)/extradural	0

*CS*, cavernous sinus; *NA*, not assessed; *NR*, not reported

^*^Some data are missing

### Radiological assessment

The literature supports the use of a complete preoperative neuro-radiological examination including magnetic resonance imaging (MRI) and computed tomography (CT) scan [5, 10, 12, 30, 37, 64, 70]. Preoperative CT can detect the presence of intratumoral calcifications, predicting a more firm and fibrous appearance at surgery. Bone CT can also add useful information about hyperostosis at the site of origin of the tumor that varies in extent from minimal to striking. Bone involvement is not predictive of tumor grade and can occur with both benign and malignant meningiomas [21]. In addition, it gives important information regarding the anatomy of the anterior clinoid process, its pneumatization, presence of a carotid-clinoid foramen, and surrounding bony structures. A contrast-enhanced MRI is now routinely performed to study tumor relationship with surrounding anatomical structures, optic canal and superior orbital fissure invasion, vascular encasement, and cavernous sinus invasion. Hyperintensity of the tumor in the T2-weighted image (T2W) is often associated with a soft consistency compared to hypointense tumors (on T2W imaging) that tends to be associated with more fibrous and firm tumors [79]. The dural vascular supply visible on T2W as a “sunburst” pattern can identify the vascular pedicle of the lesion. Gradient echo sequences (T2 GRE) are helpful to detect intratumoral calcification. MRI is also fundamental in evaluating tumor extensions to the cavernous sinus and optic canal. The constructive interference in steady state (CISS) sequence, a type of T2W gradient echo imaging, has been shown to be more effective than standard T1- and T2-weighted sequences in delineating key structures around the tumors and cerebrospinal fluid circumambient structures [26, 78]. The contrast-enhanced T1-weighted volume-interpolated breath-hold examination (VIBE) sequences have been shown to have a higher accuracy than standard sequences for predicting optic canal tumor extension with a good interrater agreement [7]. Volumetric tumor measurements are often of value to assess sequentially the indication for surgery in very small tumors and also to assess the efficacy of surgery and/or radiotherapy.

While conventional cerebral angiography can be performed in selected cases to detect major vessel encasement or displacement [5, 10, 20, 30, 37, 48, 63, 64], the same information may be obtained by less invasive radiological studies such as MR or CT angiography (MRA and CTA).The feeding arteries for clinoidal meningiomas usually come from dural branches of the internal carotid arteries or middle meningeal [62] and these lesions can be early devascularized by a skull base approach.

*The literature supports the use of a complete preoperative neuro-radiological examination including MRI, CT scan, CTA, and/or MRA. High quality T2- and T1-imaging techniques (CISS and VIBE) may be used to increase visualization of tumor extension within the optic canal and cavernous sinus. In current practice, DSA may be considered in selected cases. (Level C).*

### Neuro-ophthalmological evaluation

The large majority of patients (> 60%) have visual acuity impairment at diagnosis [19], due to the intimate relationship of the tumor with the optic nerve. The most common symptom is a slowly progressing loss of monocular visual acuity (> 80% of cases) with more than two-thirds of patients presenting with already severely impaired vision. The underlying pathophysiological mechanisms are related to chronic ischemia in addition to the mechanical compression of the nerve. Preoperative visual status, duration of symptoms, lesion size, and adherence to internal carotid artery (ICA) and its branches have been shown to be significant determinants of postoperative visual outcome. Pooled rate of visual improvement after surgery for anterior clinoidal meningiomas is observed in less than half of patients (48%, 95% CI 38.6–57.4%)[19]. This outcome is worse compared to planum sphenoidale, tuberculum/diaphragma sellae meningiomas [18, 39, 47], probably due to the intimate relationship of the tumor with the vascularization of the optic nerve and the greater risks associated with intraoperative manipulation of the optic apparatus [56, 73]. Despite the fact that the rate of optic canal invasion is less than a third (20.9%) for clinoidal meningiomas [19], lesser than that for tuberculum/diaphragma meningiomas [18, 40, 53], the rate of visual improvement is inferior in clinoidal meningiomas, possibly attesting to the higher risk of ischemic involvement of the nerve in these tumors [52]. However, the incidence of the visual deterioration after surgery remains the same for both tumor groups (4.5%, 95% CI 3.0–6%), possibly related to the improved surgical approaches and techniques [19].

*Considering the high rate of visual impairment, we recommend the use of a detailed neuro-ophthalmological examination including visual acuity, visual field examination, optic fundoscopy, and optical coherence tomography (OCT) before and usually 3 months after surgery (or earlier if there are new deficits). (Level C).*

### Surgical classification

Several classification schemes have been proposed as methods for predicting surgical outcome, though giant tumors of the clinoid process can often be challenging to classify due to the difficulty in differentiating the primary attachment from secondary extensions [2, 20, 49, 50, 55, 62, 63, 75]. Of these classifications, the Al-Mefty classification [2] has been widely accepted [9, 10, 19, 55, 60, 73]. This classification considers the tumor invasion pattern, according to its site of origin, as an indicator of resectability. It divides the ACMs into 3: group I for tumors arising proximal to the end of the carotid cistern, inferomedial surface of the ACP (no arachnoidal dissection plane between the ICA and tumor); group II for tumors arising distal to the segment of the carotid invested in the carotid cistern, arising from the lateral and superior surface of the ACP (which do have an arachnoidal plane between ICA and tumor; and group III, meningiomas that originate at the optic foramen. In group III tumors, the arachnoid membrane is present between the ICA and the tumor, but may be absent between the optic nerve and the tumor. Goel [20] and Nanda [50] proposed two similar scoring system based on anatomical, clinical, and radiological parameters. Nakamura et al. [49] divided ACMs in two groups based on cavernous sinus invasion. Pamir [55] modified the Al-Mefty classification, by adding the diameter of the tumor (< 2, 2–4, > 4 cm). These classifications based on a scoring system have the disadvantage of being complex and not user-friendly; moreover, their predictive value for tumor resectability and postoperative functional outcome has not yet been validated by larger studies. Despite the practical implication of the Al-Mefty classification with regard to surgical resectability, the distinction between the various groups is not always possible in the preoperative analysis, especially for large tumors. Nevertheless, our previous meta-analysis [19] showed that resection quality and gross total resection (GTR) rate are globally homogenous across the published studies for all Al-Mefty subgroups [19], proposing this classification for clinical and comparative use.

#### Can preoperative imaging differentiate between Al Mefty groups I and II?

The difficulty in surgically excising anterior clinoid meningiomas primarily stems from its relationship to the ipsilateral ICA and its branches especially in adventitial involvement in the absence of an arachnoidal plane. A classification system may be considered useful in surgical planning if can be reliably derived from the preoperative imaging. Several groups have analyzed preoperative radiological parameters in order to predict tumor resectability [1, 2, 5, 9, 10, 19, 20, 30, 34, 49, 50, 55, 60, 62–65, 70, 73, 75]. Nevertheless, the most modern magnetic resonance techniques and angiography do not allow, with sufficient sensitivity, the detection of an arachnoidal plane between the tumor and the artery. Most authors [9, 10, 55, 73] who adopted the Al-Mefty classification do not specify whether it is an intraoperative determination or a preoperative radiological classification. Some clearly admit that in large tumors, it is difficult to differentiate between groups I and II from preoperative images alone, and they have reclassified the lesion based on intraoperative evidence of vessel encasement [19, 60, 62].

Angiographic demonstration of direct arterial feeders from the carotid artery with narrowing of the ICA may point towards Al-Mefty Group I tumors, predicting difficulty in complete dissection off the artery. However,, a statistically significant correlation between the radiological findings and extent of resection has not been demonstrated [62]. When the ICA is encased greater than 180° and/or stenosed, the adventitia is more likely to be invaded [20, 49, 50, 63, 70]. However, Goel et al. [20] found that arterial narrowing appeared to be more related to the tumor consistency rather than invasion of the arachnoidal plane. In his original paper, Al-Mefty [2] found that an arachnoidal plane was absent only in less than 30% of the cases which had a total encased ICA. Similarly, Bassiouni et al. [5] showed that only in 11 out of 51 patients with a completely encased ICA was the tumor adherent to the vessel adventitia. Increasingly sophisticated MRI techniques, such as “high-resolution compressed-sensing T1 black-blood MRI,” offers promising potential in the context of neurovascular vessel wall imaging [23, 69, 77]. This sequence has been proven to assess extent of tumor and vessel lumen or wall infiltration, mostly for venous structures, by replacing and combining the information that were previously derived from two separate sequences 2D TOF (Time of Flight) magnetic resonance angiography and post-contrast 3D MPRAGE (Magnetization Prepared RApid Gradient Echo) T1 MRI [23]. Yin et al. demonstrated the ability of slip interface imaging (SII), a recently developed magnetic resonance elastography-based technique, to predict the degree of meningioma-brain adhesion and pial invasion, allowing for improved prediction of surgical risk and tumor resectability [80]. However, to the best of our knowledge, there is presently no literature that assesses the sensitivity of these techniques in determining the presence of an arachnoidal plane between the tumor and the arterial wall.

*The literature supports the use of anatomical classification when reporting the results of ACM surgery enabling comparison between series and different surgical approaches. The main factors determining quality of resection and postoperative complications seem to be the intimate relationship between the tumor and vessel adventitia and cavernous sinus invasion. Authors are encouraged to consider these details in tumor description. (Level C and Expert opinion).*

### Surgical technique

#### Preoperative embolization?

The utility of preoperative embolization remains controversial. There is paucity of data regarding the usefulness of preoperative embolization for clinoidal meningiomas. A recent systematic review and meta-analysis, performed by Ilyas et al. [27], showed that embolization was associated with a significant complication rate of 12% yet added no significant advantage in terms of quality of resection. Some authors have reported the use of preoperative embolization in selected cases of tumors having a significant external carotid artery supply [5, 62, 64].

#### “Vascular surgery” or “skull base” perspective?

Historically, two antithetical surgical techniques have been described for resection of an anterior clinoidal meningioma, namely, a “vascular surgery” and a “skull base” perspective. The vascular perspective consists of extensive dissection of the distal part of the Sylvian fissure to delineate and follow the middle cerebral artery toward the ICA [6]. The rationale is to keep the main artery under continuous vision while removing the tumor piecemeal, enabling safe dissection of the small perforators arising from the main arterial trunks. The main criticism of a vascular approach is that, in order to trace middle cerebral artery branches back to the main ICA trunk, the compressed Sylvian fissure needs to be opened and this carries a higher risk of stretching the arteries and of retraction injury to the brain [2]. Although some surgeons continue to use this technique, others favor a skull base approach with extensive epidural bone work along with an anterior clinoidectomy that has the advantage of expanding the surgical corridor with minimal brain retraction to achieve early optic nerve decompression and devascularization of the tumor at its dural origin [2, 34]. With this technique, the intraoperative and postoperative vascular complication rates were found to be 1.0% (95% CI, 0.4–1.6%; *P*. 0.960) and 1.9% (95% CI, 1.1–2.7%; *P*. 0.103), respectively, and new postoperative cranial nerve deficits were observed in only 5.5% of patients (95% CI, 3.6–7.5%). Mortality rate is also very low, estimated to be 1.2% (95% CI, 0.6–1.8%; *P*. 0.841) [19]. This technique is at present the most commonly used approach [19], and the different variants (standard pterional approach, fronto-orbito zygomatic approach, and suprabrow lateral subfrontal approach) can be tailored according to the anatomy and tumor size [19]. The extent of the Sylvian fissure opening and dissection will depend essentially on the encasement of the carotid artery bifurcation, A1 and M1 segments, and their branches.

#### Clinoidectomy or not?

Anterior clinoidectomy is now an integral part of the approach to the anterolateral skull base for the treatment of clinoid meningiomas, adding significant advantages including early devascularization of the meningioma, early optic nerve decompression, reducing manipulation of the visual pathway, and early identification of the ICA and optic nerve, reducing the possibility of injuring these structures [2, 15, 19, 34, 43, 66]. In addition, it could provide access to the clinoidal segment of the artery for proximal control in addition to increased access to remove tumors from the carotid cave.

An extradural anterior clinoidectomy (EAC) is the most common procedure in most published series [1, 2, 4, 10, 12, 19, 30, 34, 37, 42, 48, 62, 65, 68, 73, 75], and its variant, the intradural anterior clinoidectomy (IAC), has been performed in selected cases by a few authors [5, 49, 50, 55, 63, 64, 70]. EAC also allows the skeletonization of 270° of the optic canal allowing safer removal of the tumor in this region. Due to the lack of comparative studies and data in literature, a specific analysis of the impact of this technique on the functional outcome and the quality of surgical excision is not available [19]. The main concern with EAC lies in the supposed higher risk of vascular and nerve injuries, and the higher chances of CSF leak in case of ACP pneumatization [81]. The extended meta-analysis we previously published showed a rate of visual deterioration of 4–5%, similar between the series, regardless whether an EAC was performed or not, and previous analysis of the literature reported a lower risk of postoperative visual deterioration when an early optic nerve decompression was routinely performed [19]. The intraoperative vascular complication rates were found to be 1.0% (95% CI, 0.4**–**1.6%; *P*. 0.960) and is homogeneous among the published series regardless of the surgical technique performed. However, the rate of CSF leak with an AC (EAC or IAC) was reported in up to 8–9% of cases by few authors [2, 4, 62, 75]. Tayebi Meybodi et al. [72] proposed a 2-step hybrid technique combining the advantages of the extradural and intradural techniques while avoiding their disadvantages. The extradural phase allows early ON decompression; however, the drilling of the optic strut and removal of the tip of the ACP is performed intradurally under direct vision of the critical neurovascular structures. This technique brings considerable advantages in paraclinoidal aneurysm surgery, but needs to be evaluated on large series dealing with clinoidal meningiomas. The best surgical strategy that can maximize surgical resection and at the same time guarantee the best functional outcome is a matter of debate. Series supporting an extradural approach with clinoidectomy show visual improvements rates in the higher range of the pooled results [19]. However, this observation must be analyzed considering the initial clinical status of the population under examination. In support of this, the series of Al-Mefty et al. [2] and Verma et al. [75] largely differ, reporting a lower rate of visual improvement; however, they reported a higher percentage of patients already blind (15% in the series of Verma et al.) or with long-lasting severe visual deficit (32%) [75]. In their series, Pamir et al. [55] reported excellent results in terms of vision improvement without performing an AC. The limitations of any form of clinoidectomy are the extra time required for the approach along with the specific expertise necessary.

#### Gross total resection (GTR) in case of Al-Mefty group 1 meningioma?

The reported outcome and GTR rate vary largely among the surgical series and the existing discrepancies may be explained by the different percentages of tumors with an infraclinoid origin (Al-Mefty group I) and cavernous sinus invasion. Pooled overall GTR of the published series is achieved in 64.2% of patient (95% CI, 57.3–71%), with a large deviation of the series at both ends of the distribution [19]. This wide divergence probably reflects the heterogeneity of the various cohorts of patients in terms of cavernous sinus (CS) invasion and tumor origin. The series reporting the higher rate of GTR [2, 34, 37, 42, 55, 65] often include only a minor percentage of Al-Mefty group I tumors or those with CS invasion when compared to series with lower reported rate of GTR [4, 68]. To corroborate these data, it is of considerable interest to note that GTR rates are globally homogeneous across studies for all Al-Mefty subgroups [19]. Notably, pooled GTR is achieved in 11.8% of patient in group I (95% CI, 2.4–21.1%) and this confirms the fact that GTR is seldom achievable in cases of an infraclinoid origin of the tumor [19].

#### GTR or STR in tumors with cavernous sinus invasion?

The ideal treatment for meningiomas is total surgical excision [25]. However, GTR is not always feasible in the cranial base [45], particularly for lesions invading the CS. CS involvement, found in 1/3 of clinoidal meningiomas, is one of the main factors limiting the extent of resection. Most series on meningeal lesions involving the CS report a recurrence rate of about 60% with a subtotal resection as compared to a rate of about 5–10% in cases of total resection [16]. Despite the microsurgical achievements which followed pioneering microanatomical studies of Dolenc in the 1980s, gross total resection of tumors invading the CS is associated with a high rate (33%) of postoperative neurological deficits and mortality [14, 16, 54, 58]. Authors that report resection of the intracavernous portion of the lesion [49, 60, 62], specifically do so in cases of soft lesions. However, despite this more aggressive approach, not only are the GTR rates (42–58.9%) similar or lower than the GTR reported in series adopting a conservative strategy (64.2%) [19] but the tumor recurrence rates are only between 5 and 15% [14, 29, 60, 62], similar to the pooled average rate of recurrence found in the literature (8.9%, 95% CI, 6–11.8%, with a mean weighted follow-up of 48.1 months) [19]. To reduce the post-therapeutic neurological deficits, recent recommendations for lesions invading the CS advocate combined treatment, relying on planned subtotal resection (without intracavernous dissection and tumor removal), and adjuvant stereotactic radiosurgery (SRS) on the intracavernous residual tumor [16, 22, 36].

#### Tumor consistency affecting the GTR rate

Tumor consistency is a well-known determinant of a GTR in meningioma surgery. This assumes an important role for surgery in clinoidal meningiomas, where this factor has a large effect on resectability due to the intimate relationship of the tumor to the carotid artery, their branches, and the optic nerve. Tumors that are predominantly fibrotic and/or calcified thereby pose a significant surgical challenge and could easily be the determining factor for a subtotal resection [28].

#### Chiasmopexy or not?

The residual tumor that is left within the CS is anatomically close to the cisternal and foraminal segment of the optic nerve. With time, the distance that it is created at surgery between the residual tumor and the ON can be lost since the nerve tends to move back to its normal position. The proximity to the optic apparatus and the risk of radiation-induced optic neuropathy often prevents many surgeons from proposing SRS [13]. In addition, discriminating clear tumor margins from the visual apparatus is often challenging [46]. A simple technical solution is to place a fat graft between the ON and the tumor to maintain the distance gained at surgery and facilitates the identification of anatomic structures. This minimum safety distance created reduces the dose received by the optic nerve below 8 Gy [19]. Avoiding an oversized graft is recommended to avoid iatrogenic compression[67]. Transsphenoidal chiasmopexy has been successful reported in reversing visual degradation in cases of empty sella syndrome following treatment of pituitary tumors [24, 82] and has been used to increase the distance between the normal pituitary gland and the residual tumor, facilitating combined treatment with SRS or radiotherapy and effectively reducing the incidence of radiation injury to the normal pituitary gland [71]. However, no current consensus exists on the advantages of this technique in clinoidal meningioma surgery and its implications on the methods and timing of SRS.

*Preoperative embolization is not indicated since a skull base approach allows early identification and devascularization of the meningioma. (Level C)*

*The literature supports the use of a skull base approach with clinoidectomy with the rationale of reducing brain retraction, avoiding complications related to a large opening of the Sylvian fissure, and performing an early devascularization of the tumor and early decompression of the involved optic nerve. The extent of the approach can be tailored on the tumor size anatomy and size. (Level C).*

*Attempts at microsurgical resection of the cavernous sinus involvement are not recommended and is not justified if the resection cannot be performed without compromising the function of the cranial nerves. (Level C).*

### Upfront radiosurgery for tumor residue

Though upfront SRS provides high local tumor control small clinoidal meningiomas without extensions to the optic foramen (level III evidence) [35], larger tumors with ON contact are seldom good candidates for SRS. However, SRS represents an efficient manner to treat CS tumor extensions following surgery for the clinoid meningiomas. The efficiency of this treatment can be extrapolated from results obtained following SRS for pure cavernous sinus meningiomas. A recent report provided long-term follow-up for cavernous sinus meningiomas following SRS and showed that 61% of patients had tumor regression, 2% were unchanged, and 15% increased, after a follow-up period of 101 months after SRS [57]. Actuarial tumor control rates after upfront SRS were 98%, 93%, 85%, and 85% at the 1, 5, 10, and 15 years, respectively. Fifteen (7.5%) patients experienced permanent cranial nerve deficits without evidence of tumor progression at a median onset of 9 months (2.3–85 months). It is now well acknowledged that marginal dose prescription ranges between 12 and 15 Gy, as no improvement of local control is achieved using more than 15 Gy [31]. One of the challenges for single fraction SRS remains the anatomical location, especially the proximity of the optic pathways. SRS and particularly Gamma Knife (GK, Elekta Instruments, AB, Sweden) can address this issue, due to its very steep gradient, provided there is no contact between the lesion and the optic apparatus. In fact, a distance of 2 to 4 mm between the optic apparatus and the tumor is enough to avoid delivering more than 8 to 10 Gy to the former, while recent studies have shown that such maximal dose can be safely increased up to 12 Gy [33]. Another challenge is related to the eventual post-SRS pituitary insufficiency. It has been advocated that a ratio of stalk-to-gland radiation dose of 0.8 or more significantly increased the risk of endocrinopathy following SRS [59]. For larger tumors with major comorbidities precluding surgical resection, hypofractionated radiosurgery (HRS) (usually 25 Gy in 3 to 5 fractions) can be used even when there is close contact or when encircling the optic apparatus [41]. Fariselli et al. [41] used CyberKnife for HRS and reported high local control rates, 93% at 5 years, while avoiding radiation-induced optic neuropathy (5.1% of visual worsening). In a recent systematic review on the role of SRS and fractionated radiotherapy (FRT) in cavernous sinus meningiomas [36], local progression-free survival at 5 years after GKS, Linac SRS, and FRT were, respectively, of 93.6%, 95.6%, and 97.4% (*p* > 0.05). Progression-free survival at 10 years in this study with these techniques were 82.3%, 87.4%, and 95.5%, respectively. Nevertheless, monofractionated treatments (GKS and Linac RS) induced more tumor volume regression than FRT, SRS achieving a twice-higher rate of tumor volume regression than FRT [36].

*SRS represents an excellent method of treatment of residual tumor with the cavernous sinus following surgery for clinoidal meningiomas as it avoids the morbidity of intracavernous sinus surgery. (Level B).*

*SRS for small tumors or HRS/FRT for larger tumors can be considered as upfront alternative management for patients deemed unfit for surgery. (Level C)*

### Quality of life (QoL) and visual quality of life

The visual-related quality of life (VRQOL) needs to be analyzed separately from other neurological functional outcomes in clinoidal meningiomas, in order to assess the impact that visual impairment has on quality of life. Of the several evaluation instruments to assess VRQOL instruments proposed in literature [32], the Vision Core Measure1 (VCM1), a 10-item self-complete scale, has been proven to be an easy applicable and useful tool to the patient’s global feelings and perceptions associated with visual impairment [17]. Vu et al. [76] clearly found that non-correctable unilateral vision loss is associated with issues of safety and independent living with significantly poorer general health scores than those with normal vision in the dimensions of ‘‘social functioning’’ and ‘‘role limitation due to emotional problems’’ [44, 76]. Neil-Dwyer et al. [51] tried to correlate the disability after skull base surgery and the burden on care givers. They concluded that the most used outcome neurological scores (Glasgow Outcome Score, 36 item short-form health survey, modified Rankin scale, etc.) do not take into account more parameters such as vocational capacity, mobility, and quality of life and they might not be adequate for patients with visual disability [44, 51, 61]. Similarly, less attention has been paid to the effect that visual disability has upon patient’s career and employment [61]. Bor-Shavit et al. [8] examined the postoperative visual outcome in skull base meningiomas close to the optical pathway and found that, regardless of the cause or origin of the disability, a visual impairment in patients who are unilaterally blind or unable to drive has a major impact on the patient’s VRQOL. Unilateral impairment of VRQOL is strongly associated with impaired distant, near, and stereo vision, and equally has important vocational and occupational implications. Vision disability in working age adults confers important adverse consequences for the “health and wealth” of the patient and this should be given a priority higher than usually accorded [44, 61].

*Visual quality of life is of primary concern in clinoidal meningioma surgery and should be given a higher priority. Patients should be counselled on the potential visual functional outcomes, including a discussion on the possibility of visual deterioration. Evaluation of the health-related quality of life represents a recommended requirement in the management of patients with a clinoidal meningioma and should be assessed before and after treatment. (Expert opinion).*

## Summary of recommendations

- *We recommend the use of a complete preoperative neuro-radiological examination including MRI, CT scan, CTA, and/or MRA. High-quality T2- and high-resolution T1-imaging techniques (CISS and VIBE) may be used to increase detection of tumor extension into the optic canal and cavernous sinus. (Level C)*
- *We recommend the use of a detailed neuro-ophthalmological examination including visual acuity, visual field examination, optic fundoscopy and optical coherence tomography (OCT) before and usually 3 months after surgery (or earlier if there are new deficits). (Level C)*
- *We recommend assessing health-related QoL and VRQoL before and after treatment. (Expert opinion*
- *Patients should be counselled on the potential visual functional outcomes, including a discussion on the possibility of visual deterioration.*
- *We recommend the use of intraoperative anatomical classification when reporting the results of clinoidal meningioma surgery. Authors are encouraged to consider the relationship between the tumor and vessel adventitia and cavernous sinus invasion in describing the tumor (Level C and Expert opinion)*
- *Preoperative embolization is not indicated since a skull base approach allows early identification and devascularization of the meningioma. (Level C)*
- *We recommend the use of a skull base approach with clinoidectomy as a general rule with the rationale of reducing brain retraction, avoid complications related to a large opening of Sylvian fissure, and performing an early devascularization of the tumor and early decompression of the involved optic nerve. The extent of the skull base approach can be tailored based on the tumor anatomy and size (Level C)*
- *In the absence of an arachnoidal dissection plane between the tumor and the arterial wall (Al-Mefty Group I tumors), a GTR may not be achieved and a STR is recommended. (Level C)*
- *Attempts at microsurgical resection of the cavernous sinus extension are not recommended. (Level C)*
- *SRS for residual tumor within the cavernous sinus following surgery for clinoidal meningiomas represents an excellent method of treatment (Level B). FRT and HRT can be considered when the residual tumor maintains too small a distance from the optic nerve (level C).*
